# From circumferential to posterior-only fixation: evolving strategies for cervical fractures in ankylosing spinal disorders

**DOI:** 10.1016/j.bas.2026.107482

**Published:** 2026-10-17

**Authors:** Paul Backhaus, Nicole Lange, Alexander Lars Quiring, Bernhard Meyer, Maria Wostrack

**Affiliations:** a Department of Neurosurgery, TUM School of Medicine and Health, Technical University Munich, Germany; b Department of Neurosurgery, BG Trauma Center Murnau, Germany

**Keywords:** Spine trauma, Cervical spine fractures, Spinal ankylosing disorders, Diffuse idiopathic skeletal hyperostosis, Ankylosing spondylitis

## Abstract

**Introduction:**

Cervical fractures in patients with ankylosing spinal disorders are highly unstable injuries associated with substantial morbidity and mortality. While surgical stabilization is widely accepted, the optimal surgical strategy remains debated.

**Research question:**

To evaluate surgical strategies, neurological outcomes, morbidity, and mortality following surgical treatment of cervical fractures in patients with spinal ankylosis.

**Material and methods:**

We retrospectively analyzed 91 consecutive patients with ankylosing spinal disorders who sustained cervical spine fractures and were treated surgically between 2008 and 2025. Demographic characteristics, fracture morphology, neurological status, surgical approach, perioperative outcomes, complications, and mortality were assessed.

**Results:**

The cohort comprised 91 patients with a median age of 78 years. Low-energy trauma accounted for most injuries. Neurological deficits were present in 28.6% of patients and were significantly associated with AO type C fractures and epidural hematoma. Surgical treatment consisted predominantly of posterior fixation, with a temporal shift from combined anterior–posterior stabilization toward posterior-only navigated pedicle screw constructs. Overall complication and in-hospital mortality rates were 52.7% and 9.9%, respectively. In multivariable Firth regression, higher Charlson Comorbidity Index remained associated with in-hospital mortality, whereas age did not.

**Discussion and conclusion:**

AO type C morphology and concomitant epidural hematoma were associated with neurological compromise. Posterior long-segment fixation using navigated pedicle screws became the predominant treatment strategy, with no mechanical failures requiring revision observed in this group during the available follow-up. Additional anterior stabilization remained important when direct anterior decompression or structural anterior column reconstruction was required.

## Highlights

• Neurological deficits affected 28.6% of patients with ankylosed cervical fractures.
• AO type C fractures and concomitant epidural hematoma were associated with deficits.
• Surgical management evolved from circumferential toward posterior fixation.
• No mechanical failures occurred after posterior-only fixation during the available follow-up.
• Morbidity remained high and in-hospital mortality reached 9.9%.

## 1 Introduction

Ankylosing spondylitis (AS) and diffuse idiopathic skeletal hyperostosis (DISH) represent two distinct pathological conditions that ultimately lead to the development of an ankylosed spine. Although both culminate in a rigid spinal column, their underlying pathophysiology differs considerably. AS is a rheumatologic disorder characterized by progressive sacroiliac and facet joint ankylosis and is strongly associated with HLA-B27, whereas DISH is a non-inflammatory enthesopathy closely associated with metabolic syndrome, obesity, and diabetes mellitus leading to ossification of the intervertebral discs and anterior longitudinal ligament (Braun and Sieper, 2007; Kuperus et al., 2020).

The structural sequelae of these disorders, namely progressive ankylosis and decreased bone mineral density, create a spine that is biomechanically vulnerable (Jacobs and Fehlings, 2008; Hartmann et al., 2017). Rather than dissipating forces along flexible motion segments, the ankylosed spine behaves as a rigid “chalk stick”. As a result, even low-energy trauma can lead to severe injury by concentrating mechanical stress using fused segments acting as long lever arms. Patients with spinal ankylosis have an approximately four-to fivefold increased risk of sustaining spinal fractures compared with the general population (Young et al., 1977; Prieto-Alhambra et al., 2015). Crucially, fractures occurring in an ankylosed spine should generally be considered mechanically unstable (Lange et al., 2005).

Neurological complications are frequent in this patient cohort. Secondary fracture dislocation, as well as concomitant spinal epidural hematomas - which occur at an increased rate compared with non-ankylosed patients - are often the cause of neurological compromise (Finkelstein et al., 1999; Hanna et al., 2021).

The difficulties in diagnosing fractures in patients with AS and DISH are well known (Finkelstein et al., 1999). Reasons include the often mild mechanism of injury and the difficulty in visualizing non-dislocated fractures in the setting of AS or DISH on plain radiographs. Therefore, CT and MRI of the entire spine are recommended to rule out injury (Schwendner et al., 2021).

From a therapeutic perspective, operative stabilization is generally recommended for these injuries, as nonoperative treatment often fails to provide sufficient stability and is associated with a high risk of secondary displacement and neurological deterioration (Robinson et al., 2015; Reinhold et al., 2018). Nonetheless, the optimal surgical approach remains a subject of ongoing debate. Advocates of isolated anterior fixation emphasize the benefits of reduced surgical morbidity, shorter operative times, and potentially lower complication rates (Shetty et al., 2023). However, critics argue that anterior-only strategies often fail to provide adequate biomechanical stability in the context of long lever arms and poor bone quality and instead advocate posterior long-segment fixation or combined anterior–posterior fixation (Hartmann et al., 2017). The aim of this study was to evaluate surgical strategies, outcomes, and predictors of complications and mortality in patients with cervical spine fractures in the setting of spinal ankylosis.

## 2 Materials and methods

### 2.1 Patient selection and diagnostic criteria

We performed a retrospective study of consecutive adult patients with subaxial cervical spine fractures in the setting of spinal ankylosis who were treated between March 2008 and July 2025 at a tertiary referral university spine center. Eligible patients were required to be aged ≥18 years, have a traumatic subaxial cervical osseoligamentous injury, and demonstrate spinal ankylosis, defined as bridging across at least four contiguous vertebral bodies. AS or DISH was assigned based on either a documented pre-existing diagnosis or retrospective review of the available imaging. DISH was defined based on the criteria of Resnick and Niwayama, including flowing anterolateral ossification across at least four contiguous vertebral bodies with relative preservation of intervertebral disc height and without extensive sacroiliac or apophyseal joint ankylosis. AS was classified based on the modified New York criteria, with radiographic sacroiliitis as the defining feature and syndesmophytes or posterior-element ankylosis considered supportive findings (Resnick and Niwayama, 1976; Linden et al., 1984). Patients were excluded if they had no subaxial cervical osseoligamentous injury or did not meet the predefined criteria for spinal ankylosis. All consecutive patients meeting these criteria during the study period were included. A total of 91 patients were included in the analysis.

### 2.2 Surgical treatment

Posterior fixation was performed using the neon3 occipitocervical screw-rod-system (Ulrich medical, Ulm, Germany) via a midline open approach. Pedicle screws were placed using intraoperative 3D navigation (Brainlab, Munich, Germany). Alternatively, lateral mass screws were placed under fluoroscopic guidance. When an anterior approach was used, a standard left-sided cervical approach was performed. Interbody or vertebral-body reconstruction was performed using cage systems available during the respective treatment period, including Cerv-X interbody cages (Ulrich Medical, Ulm, Germany) and ATHLET vertebral body replacement devices (Signus Medizintechnik, Alzenau, Germany). Anterior plating was performed using the VENTURE anterior cervical plate system (Medtronic, Minneapolis, MN, USA).

Surgical treatment was categorized as posterior only, anterior only, posterior followed by anterior surgery, or anterior followed by posterior surgery. A second approach that had not been documented as part of the initial treatment strategy was classified as unplanned. The interval between procedures was calculated only for staged operations; procedures completed during the same anesthetic session were excluded from the interval calculation.

### 2.3 Outcomes and statistical analysis

Primary outcomes included duration of surgery, intraoperative blood loss, length of hospital stay, and complication and revision rates. Volumetric bone mineral density was assessed opportunistically from non-contrast-enhanced CT examinations using SpineQ® software (version 1.0; Bonescreen GmbH, Munich, Germany). The analyzed vertebral levels varied according to the available CT coverage and successful automated identification of a valid vertebral centroid. Vertebrae with fractures, marked structural abnormalities, focal osseous pathology, or other changes precluding reliable measurement were excluded. Automated vertebral segmentations and measurements were visually inspected for plausibility, and technically inadequate measurements were excluded.Bone density was categorized as normal at >120 mg/cm^3^, osteopenic at 80–120 mg/cm^3^, and osteoporotic at <80 mg/cm^3^.

Statistical analyses were conducted using R statistical software (R version 4.5.0 [2025-04-11]). Continuous variables were compared using the Wilcoxon rank-sum test or Kruskal-Wallis test, as appropriate. Categorical variables were analyzed using Fisher's exact test. Associations with in-hospital mortality were assessed using Firth penalized logistic regression because of the limited number of events. The multivariable model was restricted to age and the non-age-adjusted Charlson Comorbidity Index; age was modeled per 5-year increase and CCI per one-point increase. Odds ratios are reported with profile penalized likelihood 95% confidence intervals. Missing data were not imputed; analyses were performed using available cases. A two-sided p < 0.05 was considered statistically significant. This study was reported in accordance with the Strengthening the Reporting of Observational Studies in Epidemiology (STROBE) guidelines for observational cohort studies.

## 3 Results

### 3.1 Patient and injury characteristics

Baseline characteristics of the study population are displayed in Table 1. A total of 91 patients with fractures in the setting of spinal ankylosis were included. The cohort consisted of 80 men (87.9%) and 11 women (12.1%), with a median age of 78 years (IQR 66–84).

**Table 1:** Baseline characteristics of the study population. Median (Q1, Q3), n (%). *Mechanism of injury was unavailable for one patient. Bone mineral density data were unavailable for three patients.

Characteristic	N = 91
Age (years)	78 (66, 84)
Sex	
Female	11 (12%)
Male	80 (88%)
ASA Score	
I	3 (3.3%)
II	41 (45%)
III	42 (46%)
IV	4 (4.4%)
V	1 (1.1%)
Age-adjusted Charlson Comorbidity Index	4 (3, 6)
Mechanism	
Bicycle accident	5 (5.6%)
Fall down stairs	12 (13%)
Ground-level fall	63 (70%)
Recurrent falls	3 (3.3%)
Motor vehicle accident	7 (7.7%)
Fracture morphology	
B3	82 (90.1%)
C	9 (9.9%)
Epidural hematoma	10 (11%)
Neurological deficit	26 (29%)
Bone Mineral Density [mg/cm^3^]	76.3 (53.6–99.4)
Osteoporosis	46 (52.3%)
Osteopenia	28 (31.8%)
Neurological status at admission	
AIS A	1 (1.1%)
AIS B	2 (2.2%)
AIS C	6 (6.6%)
AIS D	10 (11%)
AIS E	65 (71%)
Radicular injury	7 (7.7%)

Low-energy trauma was the predominant mechanism. Ground-level falls were the most common mechanism of injury, accounting for 63 cases (69%), followed by stair falls in 12 patients (13%). Less common mechanisms included motor vehicle accidents (n = 7), bicycle accidents (n = 5) and recurrent falls (n = 3). All patients were examined with whole-spine CT (100%) and MRI, when possible (98%). Most fractures were hyperextension injuries (AO B3, n = 82, 90.1%), while translational injuries were observed in 9 patients (AO C, n = 9, 9.9%). The most affected spinal segment was C6/7 (n = 23), followed by C5/6 and C6 (n = 17 each) (see Fig. 1). Secondary injuries in the thoracic and/or lumbar spine were present in 18 patients (19.8%).

**Fig. 1:**
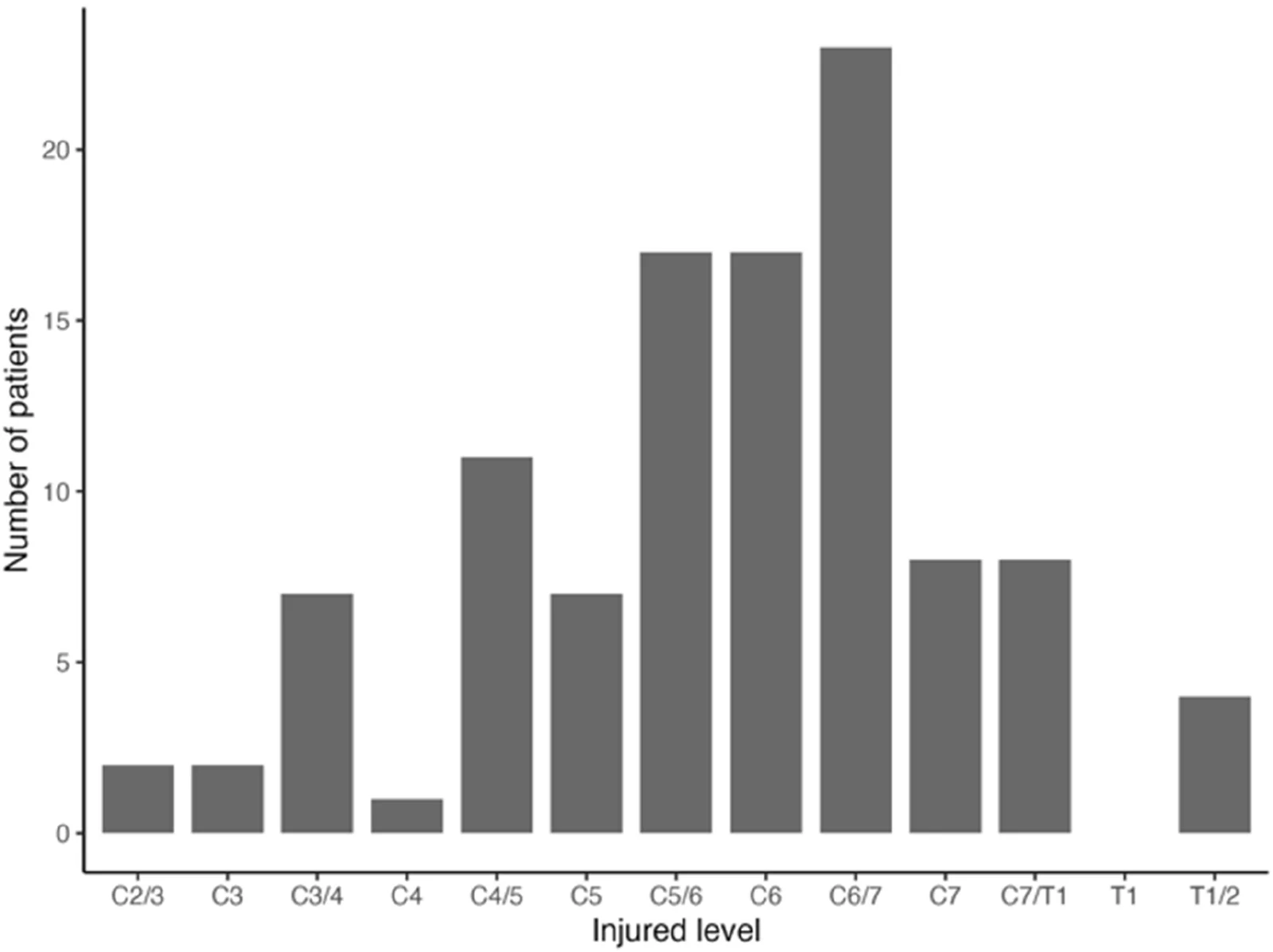
Anatomical distribution of cervical spine fractures in patients with spinal ankylosis. Multiple levels could be involved in individual patients.

Of the 91 included patients, 41 (45.1%) were classified as having AS and 50 (54.9%) as having DISH. Among patients with AS, the diagnosis had been established before the index trauma in 32 of 41 patients (78.0%), whereas 9 patients (22.0%) were classified based on the trauma imaging. None of the patients with DISH had a previously documented diagnosis; all 50 cases were identified from imaging obtained during the fracture work-up.

Bone mineral density assessment was available in 88 of 91 patients (96.7%), with a median of 76.3 mg/cm^3^ (IQR 53.6–99.4). Osteoporosis was present in 46 patients (52.3%), osteopenia in 28 (31.8%), and normal bone density in 14 (15.9%).

Neurological compromise was present in 26 patients (28.6%) and was significantly associated with both fracture morphology and the presence of epidural hematoma. Among patients with AO type B3 fractures, 24.4% (n = 20/82) presented with a neurological deficit compared to 66.7% of those with AO type C fractures (n = 6/9), representing a statistically significant difference (p = 0.015, OR 6.2). Similarly, neurological deficits were more frequent in patients with epidural hematoma occurring in 80.0% (n = 8/10), compared to 22.2% (n = 18/81) in those without (p < 0.001, OR 14.0). Of those presenting with neurological deficit, most patients presented with incomplete spinal cord injury (AIS D: 10, AIS C: 6, AIS B: 2), whereas 1 patient had complete spinal cord injury. Seven patients presented with isolated radicular deficits at the injured segment; these patients were categorized separately and not included in the AIS classification above.

### 3.2 Surgical treatment and operative characteristics

Surgical treatment was performed in all patients. Posterior-only fixation was performed in 63 patients (69.2%), isolated anterior fixation in 3 (3.3%), and combined anterior–posterior stabilization in 25 (27.5%). Posterior-only stabilization was generally selected when adequate reduction and long-segment fixation could be achieved without a need for direct anterior decompression or anterior column reconstruction.

Among the combined procedures, posterior fixation was performed first in 13 patients and anterior fixation first in 12. In the posterior-first group, the anterior component had been planned from the outset in 7 patients; in the remaining 6, anterior supplementation was added after posterior stabilization because of a persistent anterior fracture gap requiring additional anterior column reconstruction. All posterior-first procedures were staged, with a median interval of 4 days (IQR 3–7) to anterior surgery. In the anterior-first group, the combined strategy had been planned in 11 of 12 patients. The anterior component was used for direct decompression, anterior column reconstruction or initial anterior exploration to confirm and directly assess the injury. Two procedures were completed during the same anesthetic session and 9 as staged procedures, with a median interval of 6 days (IQR 2–7) to posterior stabilization; in 1 patient, the planned posterior stage could not be performed because the patient died before the scheduled second-stage procedure.

All patients with symptomatic epidural hematoma (n = 8) were treated with dorsal or dorsoventral instrumentation, laminectomy and hematoma evacuation.

Constructs spanned a median of 4 motion segments overall (IQR 3–5). Posterior-only constructs spanned a median of 5 segments (IQR 4–5), compared with 2 segments for anterior-only constructs (IQR 1.5–2.5) and 4 segments for combined constructs (IQR 3–6). Posterior fixation extended a median of 2 segments above the most cranial injured level (IQR 2–2) and 2 segments below the most caudal injured level (IQR 2–3).

Temporal analysis revealed a shift in surgical practice with combined anterior–posterior constructs used more frequently during the earlier years and posterior-only pedicle screw-based constructs becoming the predominant strategy during the later period (Fig. 2).

**Fig. 2:**
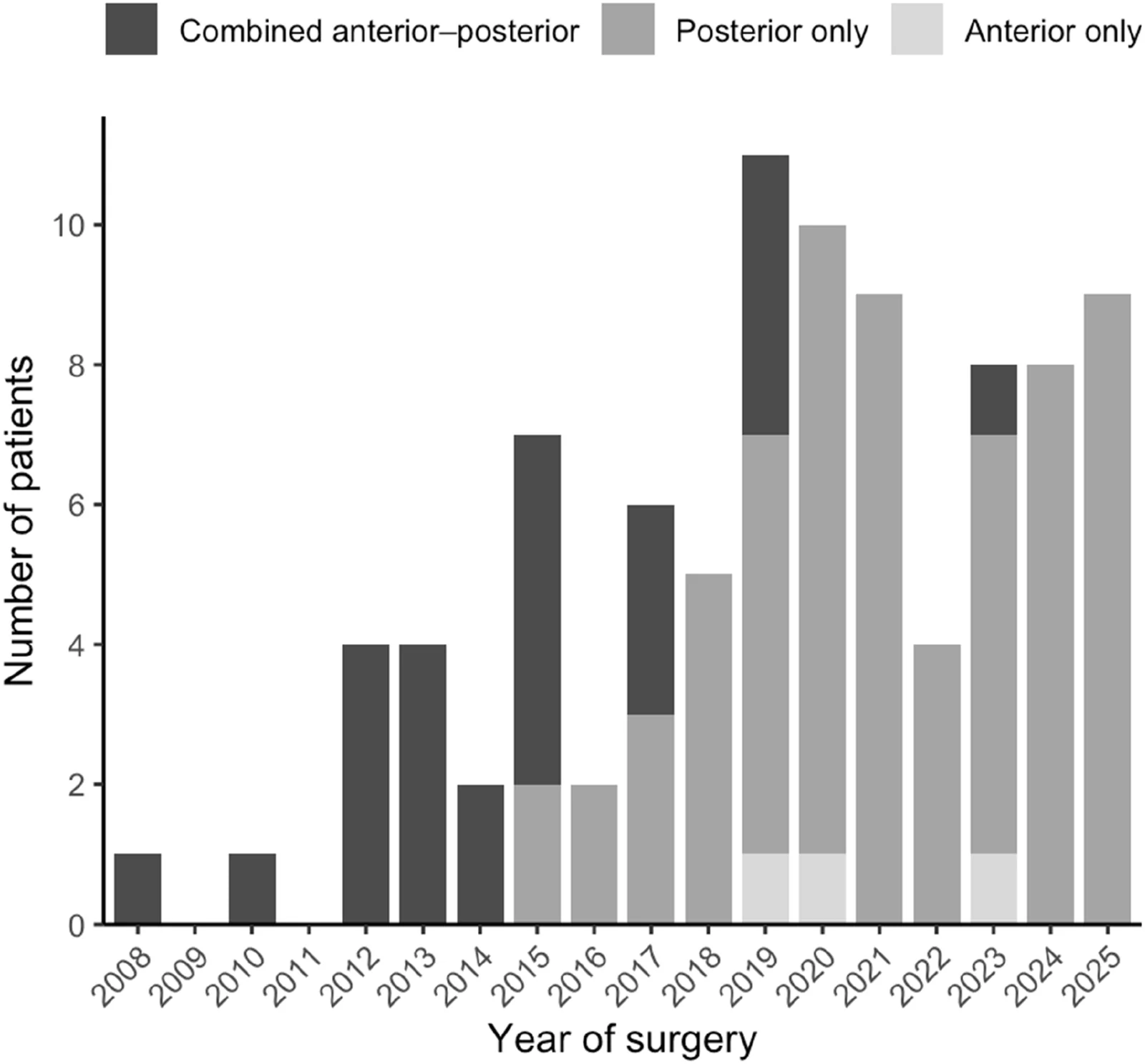
Temporal distribution of surgical approaches. Annual numbers of patients undergoing posterior-only fixation, combined anterior–posterior stabilization, or anterior-only fixation between 2008 and 2025.

**Fig. 3:**
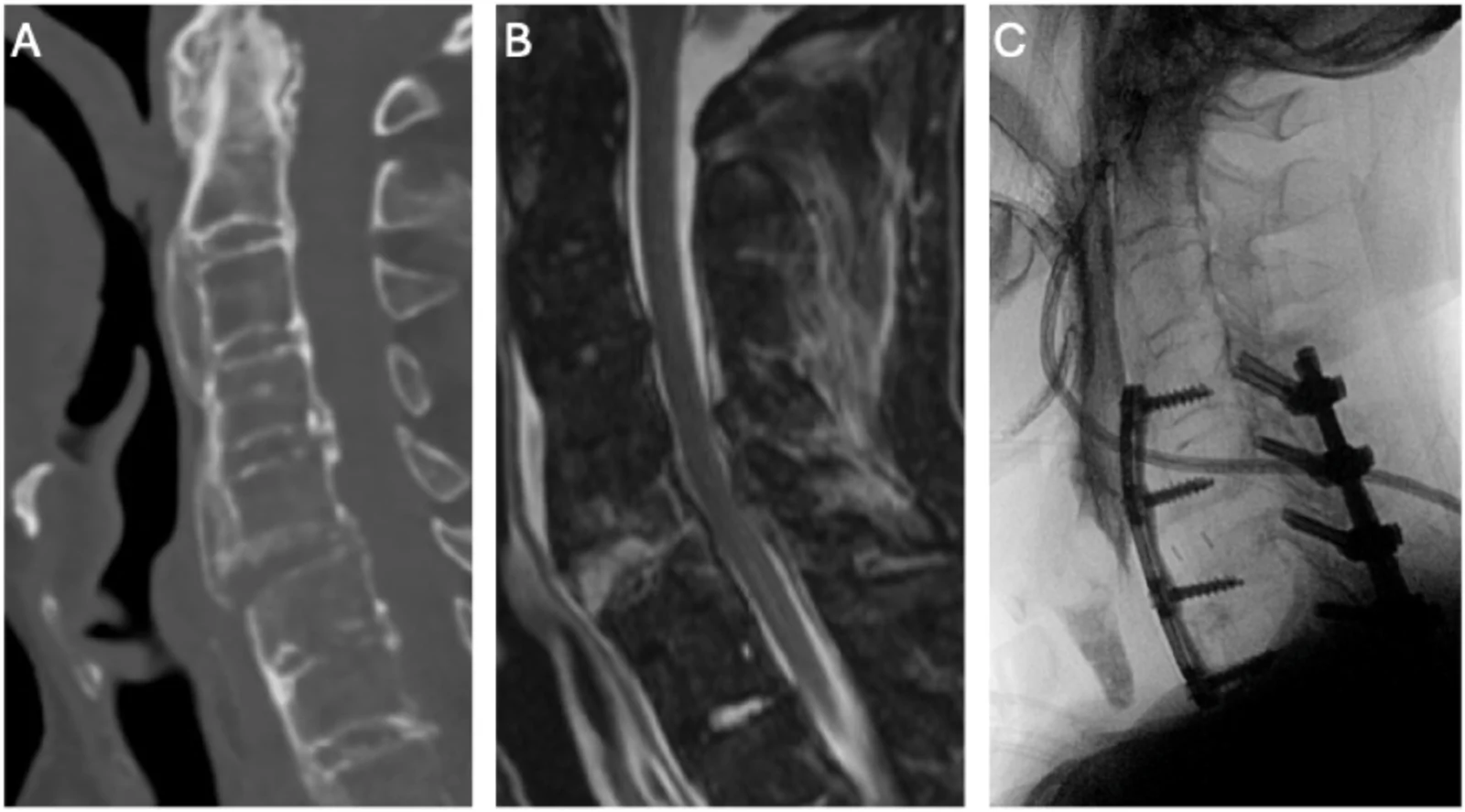
Dorsoventral fixation for a B3 Fracture at C5/6. A Preoperative CT scan showing AO B3 fracture at C5/6 level. B Preoperative MRI showing the fracture as well as edema in the nuchal ligaments, indicative of injury to the anterior and posterior column. C Postoperative X-Ray showing dorsoventral fixation using anterior plating as well as posterior fixation with lateral mass screws. **Exemplary case description (2013)** – Fig. 3**:** An 80-year-old patient presented to the emergency department with neck pain following a low-energy fall. CT imaging demonstrated a hyperextension injury at the C5/6 level. Neurological examination was unremarkable. Surgical management consisted of anterior cervical discectomy and fusion, followed two days later by posterior stabilization with lateral mass screws. The postoperative course was complicated by pneumonia, respiratory failure, and dysphagia requiring tracheotomy, which resulted in a prolonged hospitalization. The patient was discharged to a rehabilitation facility after 31 days.

**Fig. 4:**
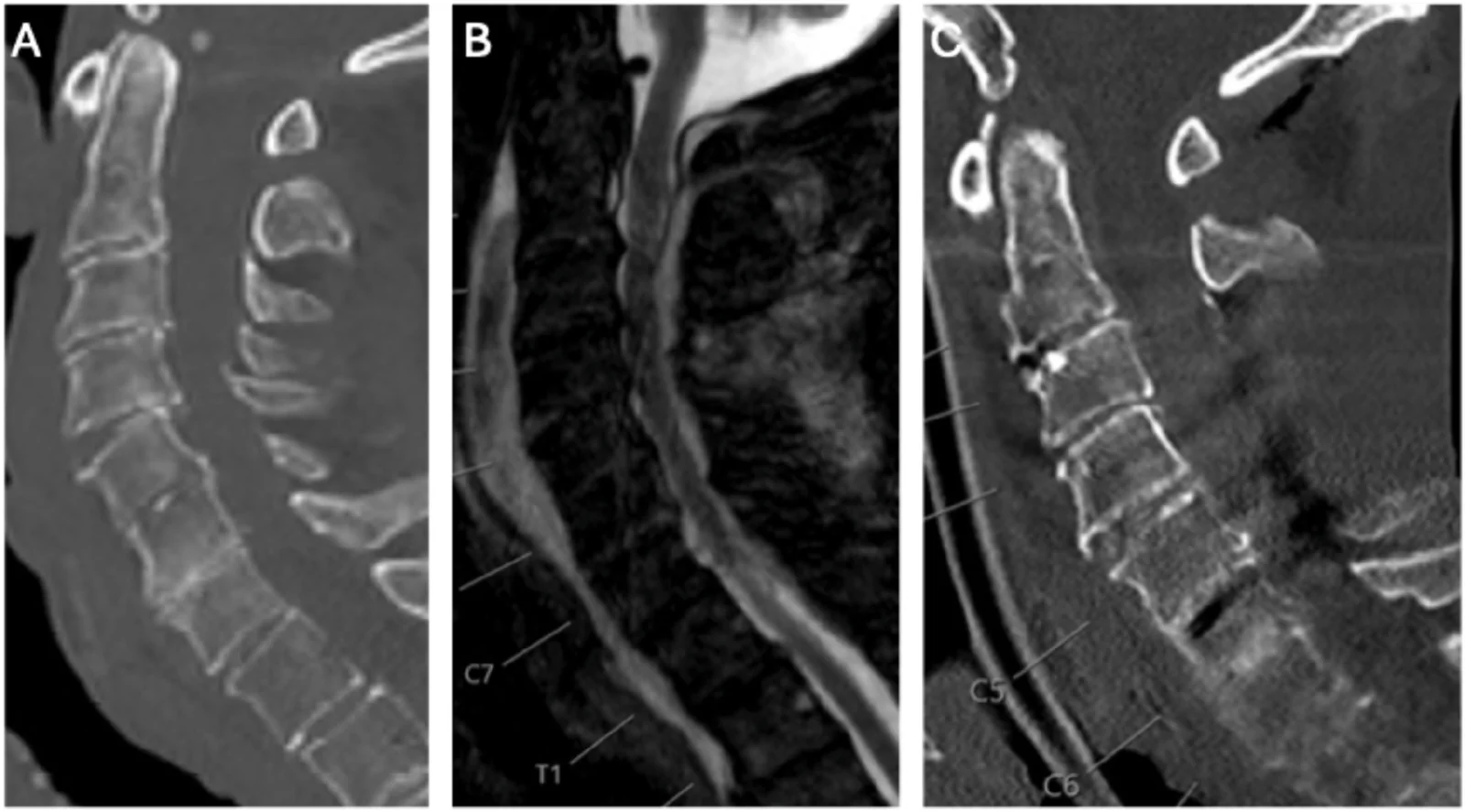
Isolated posterior fixation for a C4/5 fracture with concomitant spinal epidural hematoma. A Preoperative CT scan showing a fracture at C4/5 disc level with failure of the anterior tension band. B Preoperative MRI showing the C4/5 fracture, a concomitant prevertebral hematoma, a spinal epidural hematoma extending from C1 to C5, and edema of the posterior ligaments, indicating failure of both the anterior and posterior tension bands. C Postoperative CT scan showing adequate reduction of the fractured segment and decompression via C3–6 laminectomy. **Exemplary case description (2025)** – Fig. 4: A 93-year-old patient presented to the emergency department after a syncopal episode with subsequent fall from a chair, reporting severe neck pain. On examination, proximal paraparesis of the upper extremities was noted with an MRC grade of 2–3/5. Imaging revealed a fracture at the C4/5 level with concomitant epidural hematoma and spinal cord compression. The patient underwent surgical stabilization with navigated pedicle screws at C3–C6, combined with decompression and hematoma evacuation via C3–C6 laminectomy. The patient experienced partial neurological improvement but remained classified as AIS D at discharge. The clinical course was complicated by pneumonia and pleural effusion, requiring intensive care for nine days. The patient was discharged to a rehabilitation facility after 25 days.

**Fig. 5:**
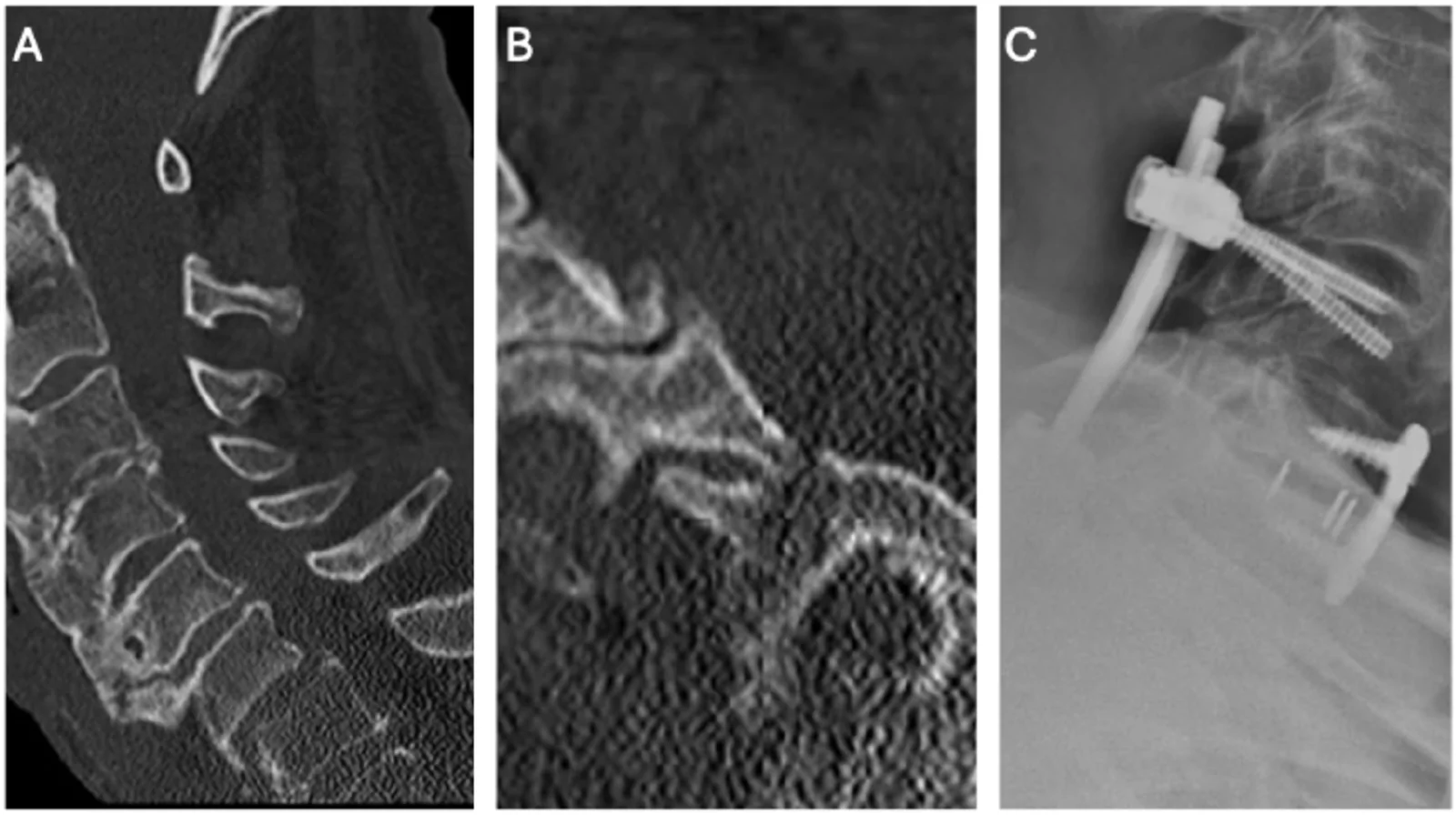
Supplementary posterior fixation after isolated anterior stabilization. A: Preoperative CT scan demonstrating anterolisthesis of the injured segment C6/7. B: Preoperative CT scan demonstrating the fracture of the facet joint. C: Lateral X-ray following posterior fixation. **Exemplary case description (2019) –**Fig. 5: A 75-year-old patient sustained a unilateral C6/7 facet dislocation with concomitant disc injury after a fall and presented with mild triceps paresis. Following anterior fixation, the patient was discharged home. Two months later, progressive mechanical neck pain and bilateral forearm dysesthesia raised clinical concern for persistent instability despite the absence of documented radiographic displacement. The patient therefore underwent supplementary posterior fixation, after which the neck pain improved substantially.

The overall median time to surgery was 3 days. In contrast, patients presenting with neurological deficits underwent surgical intervention considerably earlier, with a median time of 7 h following initial imaging.

Median blood loss was lowest in anterior-only procedures (200 mL [100–300 mL]) compared with posterior-only (800 mL [500–1500]) and combined anterior–posterior approaches (800 mL [300–1700]); however, this difference did not reach statistical significance. The small number of anterior-only procedures should be considered when interpreting this finding. In contrast, operative duration differed significantly between surgical approaches (p = 0.011). Post hoc analysis demonstrated that anterior-only procedures (70 min [58–137]) were significantly shorter than both posterior-only (228 min [188–291]) and combined anterior–posterior procedures (246 min [212–316]), whereas no significant difference was observed between posterior-only and combined anterior–posterior approaches. Median length of hospital stay did not differ between approaches, although slightly longer stays were observed following combined anterior–posterior procedures (15 days [12–24]) compared with posterior-only (11 days [5–18]) and anterior-only procedures (13 days [4–22]).

### 3.3 Perioperative and in-hospital outcomes

In-hospital postoperative complications occurred in 34 of 63 patients treated posteriorly (54.0%), 13 of 25 treated with a combined anterior–posterior approach (52.0%), and 1 of 3 treated anteriorly (33.3%), with no difference between approaches (Fisher's exact test, p = 0.858, Table 2). The most common surgery-related complications were surgical-site infection (n = 8), fascial dehiscence (n = 5), and postoperative hemorrhage (n = 1). Within 30 days of surgery, revision was required in 9 of 63 patients treated posteriorly (14.3%), 4 of 25 treated with a combined anterior–posterior approach (16.0%), and none of the 3 patients treated anteriorly. The overall revision rate did not differ between surgical approaches (Fisher's exact test, p = 1.000). Overall, 13 patients underwent 20 short-term revision procedures, most commonly for surgical-site infection or fascial dehiscence. Other indications included postoperative hemorrhage and individual implant- or construct-related complications.

**Table 2:** Postoperative complications and short-term revisions according to surgical approach. Values are presented as n (%). SSI, surgical-site infection. Comparisons were performed using Fisher's exact test.

	Posterior-only (n = 63)	Combined anterior–posterior (n = 25)	Anterior-only (n = 3)	p
Any complication	34 (54%)	13 (52.0%)	1 (33.3%)	0.858
SSI	6 (9.5%)	2 (8.0%)	0 (0.0%)	
Fascial dehiscence	4 (6.3%)	1 (4.0%)	0 (0.0%)	
Postoperative Hemorrhage	0 (0.0%)	1 (4.0%)	0 (0.0%)	
Any medical complication	23 (36.5%)	9 (36.0%)	1 (33.3%)	
Revision < 30 days	9 (14.3%)	4 (16.0%)	0 (0.0%)	1.000
Death	5 (7.9%)	4 (16.0%)	0 (0.0%)	

A total of 817 posterior screws were placed, including cervical lateral mass screws, cervical pedicle screws, and upper thoracic pedicle screws. Eighteen screws were classified as malpositioned, corresponding to an overall malposition rate of 2.2%. Among 464 cervical pedicle screws, 16 were malpositioned (3.4%). One patient underwent revision surgery for symptomatic screw malposition with radiculopathy. Vertebral artery injury occurred in three patients; all were treated with coil occlusion, and none developed a resulting neurological deficit.

Perioperative neurological deterioration occurred in three patients. In one case, foraminal stenosis after posterior fixation caused radicular injury that was successfully treated with revision surgery. In another case, fracture dislocation occurred after the anterior stage of a planned combined anterior–posterior procedure in a neurologically intact patient, resulting in an AIS C injury; immediate reduction and posterior fixation were then performed. A third patient developed worsening tetraparesis after anterior-only surgery and was managed conservatively.

The most common medical complications were pneumonia (n = 20), acute kidney injury (n = 8) and pleural effusion (n = 6). Less frequent events included respiratory failure, delirium, pulmonary embolism, bloodstream infection, urinary tract infection, acute coronary syndrome or heart failure, and pneumothorax.

Overall, nine patients (9.9%) died during the hospital stay. Patients who died were older than survivors and had a higher Charlson Comorbidity Index in unadjusted comparisons (p = 0.040 and p < 0.001, respectively). In univariable Firth regression, age showed a borderline association with mortality (OR 1.42 per 5-year increase, 95% CI 0.99–2.28; p = 0.055), whereas higher CCI was associated with mortality (OR 1.64 per point, 95% CI 1.22–2.30; p = 0.001). After mutual adjustment, CCI remained associated with in-hospital mortality (adjusted OR 1.53 per point, 95% CI 1.13–2.17; p = 0.006), whereas age was not (adjusted OR 1.24 per 5-year increase, 95% CI 0.83–2.07; p = 0.308).

At discharge, 33.0% of patients were able to return home, whereas the majority required further inpatient care, including rehabilitation (29.7%), transfer to another hospital (24.2%) or institutional care (4.4%).

### 3.4 Follow-up

Follow-up was available for 37 patients. Mean duration of follow-up was 25.2 months.

Five patients underwent a total of nine revision procedures more than 30 days after surgery for surgical-site infection, fascial dehiscence, or delayed mechanical instability. These involved 3 of 63 patients treated posteriorly (4.8%), 1 of 25 treated with a combined anterior–posterior approach (4.0%), and 1 of 3 treated anteriorly (33.3%). The overall rate of revision beyond 30 days did not differ between approaches (Fisher's exact test, p = 0.176).

Across the entire postoperative course, mechanical failure requiring unplanned revision occurred in two patients, both after anterior fixation. One patient developed screw pullout with dislocation after the anterior stage of a planned combined anterior–posterior procedure, resulting in an AIS C injury and requiring immediate reduction and posterior fixation. The second patient, treated with anterior-only fixation, developed delayed instability two months after surgery and underwent subsequent revision. No mechanical failures occurred after posterior-only fixation.

Radiographic follow-up was available in 22 patients (24.2%), including 13 of 63 patients treated with posterior-only fixation (20.6%), 7 of 25 treated with combined anterior–posterior stabilization (28.0%), and 2 of 3 treated with anterior-only fixation (66.7%). Median radiographic follow-up was 5 months (IQR 1–22), 9 months (IQR 6–36.5), and 15 months (IQR 9–21), respectively. Follow-up imaging comprised CT in 17 patients and conventional radiographs in 5. Apart from the previously described anterior-only construct failure at 2 months, no secondary displacement or implant failure was observed. Five CT examinations obtained within 2 months showed no osseous fusion, as expected at this early interval. Among 12 patients with imaging at 6 months or later, 10 underwent CT, demonstrating fusion in 9 and partial fusion in 1. In the remaining 2 patients, only conventional radiographs were available, precluding reliable assessment of osseous fusion.

Neurological follow-up was available for eight patients with preoperative neurological deficits, including five treated with a combined anterior–posterior approach, two treated posteriorly, and one treated anteriorly. Clinical improvement or regression of neurological deficits was documented in all patients. Five of six patients with radicular or segmental deficits showed regression of paresis, while the remaining patient with a sensory C8 syndrome was documented as neurologically stable at follow-up. Among two patients with central cord syndrome treated with a combined anterior–posterior approach, gait ataxia improved in one patient with pre-existing deficits following poliomyelitis, whereas the other improved but retained distal weakness of the left upper extremity (MRC grade 4/5).

## 4 Discussion

Surgical treatment for fractures in the ankylosed spine is widely regarded as the standard of care, given the inherent instability of these injuries and the high risk of neurological deterioration (Reinhold et al., 2018). Nevertheless, the optimal surgical approach remains a matter of debate. Several techniques have been described, ranging from isolated anterior stabilization to combined anterior–posterior constructs, but no clear consensus has been established. The DGOU recommends combined anterior–posterior instrumentation for unstable B- and C-type cervical injuries in ankylosing spinal disorders, reflecting concerns that a single approach may provide insufficient stability. However, when a safe anterior approach or secure anterior fixation cannot be achieved, a longer posterior construct using lateral mass and/or pedicle screws is recommended as an alternative (Reinhold et al., 2018).

In our cohort, we observed a clear temporal shift in surgical strategy. Earlier in the study period, combined anterior–posterior fixation using lateral mass screws was frequently employed. More recently, however, posterior-only fixation with navigated pedicle screws has become the predominant strategy at our institution. In our cohort, no revision for mechanical failure was required after posterior-only fixation during the available follow-up, and additional anterior stabilization was not routinely necessary. When an additional anterior procedure was required, intraoperative reduction had remained incomplete, leaving a residual fracture gap that was considered unlikely to heal without anterior column reconstruction. In most hyperextension injuries, however, adequate reduction could be achieved by applying slight flexion in the Mayfield clamp, thereby avoiding circumferential stabilization.

The role of anterior-only fixation remains controversial. Since most fractures in the context of ankylosis represent hyperextension injuries, reconstruction of the anterior tension band by long-segment plating seems reasonable. However, after initial publications showed failure rates of up to 50%, the approach was quickly abandoned by most surgeons (Einsiedel et al., 2006). Still, some authors have advocated for isolated anterior stabilization in selected cases, arguing that adequate stability can be achieved with fewer perioperative complications (Shetty et al., 2023; Volker Bühren, 2013; Chen et al., 2024). Patient selection, such as isolated failure of the anterior column, and technical nuances, such as bicortical screw placement might play a crucial role in success.

Currently, most surgeons choose an isolated posterior or combined approach to treat these fractures (Hartmann et al., 2017). Although circumferential fixation provides the greatest stability, the necessity of an additional anterior procedure is questionable. While some studies report good results without implant failure using isolated lateral mass screw fixation, others have observed implant failures and therefore recommended supplementary anterior stabilization (Lange et al., 2005; Robinson et al., 2015). Pedicle screw instrumentation in the subaxial cervical spine, first pioneered by Abumi, has gained increasing popularity in recent years (Abumi et al., 1994; Cechin et al., 2023; Rienmuller et al., 2017). Compared with lateral mass screws, pedicle screws provide markedly greater pull-out strength and can be inserted safely despite the proximity of neurovascular structures (Jones et al., 1997). Previous studies have reported satisfactory mechanical stability with isolated posterior pedicle screw fixation in ankylosing spondylitis, consistent with the absence of mechanical failure after posterior-only fixation during the available follow-up in our cohort (Yan et al., 2017). These mechanical advantages of long-segment posterior fixation, however, must be weighed against the morbidity associated with extensive posterior exposure.

Wound-related complications are of particular concern in this population. Long posterior cervical constructs require extensive nuchal soft-tissue dissection in patients who are often elderly, multimorbid, immobilized, and dependent on prolonged postoperative care, all of which may impair wound healing. In our cohort, surgical-site infection and fascial dehiscence were the most frequent surgery-related complications and accounted for a substantial proportion of early revisions. Minimally invasive navigated cervical pedicle screw fixation may reduce posterior soft-tissue disruption and wound morbidity; however, current evidence is limited to technical series and observational comparative studies, and randomized comparisons with conventional open fixation are lacking (Schaefer et al., 2011; Coric and Rossi, 2022). Its applicability may also be limited in displaced injuries requiring open reduction, decompression, or direct evacuation of an epidural hematoma.

This study has several limitations. First, its retrospective single-center design introduces the risk of selection and information bias and may also limit the generalizability of these findings to centers with different patient populations, surgical expertise, or instrumentation strategies. Second, surgical management evolved considerably over the study period, with a transition from combined anterior–posterior fixation, often using lateral mass screws, to predominantly posterior fixation using navigated pedicle screws. Consequently, the treatment groups were not directly comparable, and observed differences in outcomes may partly reflect temporal changes in surgical technique, patient selection, and perioperative care. Third, only a small number of patients underwent isolated anterior fixation, limiting meaningful comparison between surgical approaches. Finally, follow-up data were available for only a subset of patients and were neither standardized nor uniformly long term. Neurological outcomes therefore primarily reflect the early postoperative course, while sustained neurological recovery, fusion, late mechanical failure, and the comparative long-term effectiveness of the different surgical approaches could not be reliably assessed. Nevertheless, the present cohort represents one of the larger single-center series of surgically treated cervical fractures in patients with ankylosing spinal disorders and provides valuable insight into evolving surgical practice in this challenging patient population.

## 5 Conclusions

Cervical fractures in patients with ankylosing spinal disorders are severe injuries associated with frequent neurological deficits, perioperative complications, and substantial mortality. AO type C morphology and epidural hematoma were closely associated with neurological compromise, highlighting the importance of early recognition and timely surgical management. Over the study period, posterior long-segment fixation using navigated pedicle screws became the predominant strategy, and no mechanical failures were observed in this group during the available follow-up. Additional anterior stabilization remained important when direct decompression or reconstruction of the anterior column was required. Owing to the retrospective design and limited follow-up, the present findings primarily reflect short-term outcomes and do not establish the long-term superiority of any surgical approach.

## Ethics declaration

The authors have NOT obtained informed consent from participants or their legal representatives. The requirement for individual informed consent was waived by the Ethics Committee because of the retrospective study design.

This study was performed in compliance with relevant laws, regulatory frameworks and guidelines where the research took place. This study was approved by the Ethics Committee at TUM School of Medicine and Health, Technical University of Munich. (Approval No. 2022-642-S-KH)

## Ethics approval

This was a retrospective observational study. The Research Ethics Committee of the Technical University of Munich confirmed that formal ethical approval was not required.

## Consent for publication

The requirement for written consent for publication was waived by the Research Ethics Committee of the Technical University of Munich.

## Data availability

The datasets generated and analyzed during the current study are available from the corresponding author on reasonable request.

## Declaration of generative AI and AI-assisted technologies in the manuscript preparation process

During the preparation of this work, the authors used ChatGPT (OpenAI, San Francisco, CA, USA) to assist with language editing, grammar correction, and manuscript readability. The authors reviewed and edited the output as needed and take full responsibility for the content of the published article.

## Funding

No external funding was received for this study.

## Declaration of competing interest

The authors declare that they have no known competing financial interests or personal relationships that could have appeared to influence the work reported in this paper.
